# The impact of a value-based hospital-community-family linkage program on blood glucose management and hypoglycemia in elderly diabetic patients: a randomized controlled trial

**DOI:** 10.3389/fmed.2025.1656364

**Published:** 2025-11-19

**Authors:** Xiaojing Zhuang, Haiyan Ding, Fafa Yu, Maoying Wang, Yuan Chen, Jiong Wu

**Affiliations:** 1 Department of Endocrinology, Zhangjiagang Sixth People’s Hospital, Suzhou, China; 2 Department of Rheumatology and Immunology, Changzhou Affiliated Hospital of Nanjing University of Chinese Medicine, Changzhou, China

**Keywords:** elderly, diabetes mellitus, blood glucose, hypoglycemia, personalized health management

## Abstract

**Trial design:**

The management of elderly type 2 diabetes (T2DM) presents significant challenges, with the risk of hypoglycemia being particularly prominent. This risk severely impacts patients’ quality of life and health outcomes. This study aims to evaluate the impact of a hospital-community-family linkage program based on value-based medicine principles and integrated with information technology on blood glucose management and hypoglycemic events in elderly T2DM patients.

**Methods:**

This study was a randomized controlled trial conducted between September 2023 and September 2024. A total of 254 elderly patients with T2DM admitted to the Zhangjiagang Sixth People’s Hospital were randomly assigned to either the linkage program group (*n* = 138) or the conventional management group (*n* = 116). The linkage program group received a 6-months structured intervention that integrated personalized health management records, regular follow-ups conducted through telemedicine platforms, and community education facilitated by digital tools. The conventional management group received standard health education, dietary guidance, and medication advice. The primary outcome measure was the incidence of hypoglycemic events. Secondary outcomes included fasting plasma glucose (FPG), 2-hour postprandial glucose (2hPG), self-efficacy in diabetes management, average daily dose of antidiabetic medications, and anxiety levels.

**Results:**

The linkage program group demonstrated significant reductions in the average number of hypoglycemic events (3.81 vs. 4.13, *P* = 0.015) and required lower doses of antidiabetic medications. Improvement in fasting plasma glucose (6.93 vs. 7.46 mmol/L, *P* = 0.013) was observed. In addition, participants reported reduced anxiety levels (88.41% low anxiety vs. 73.28%, *P* = 0.005).

**Conclusion:**

The value-based hospital-community-family linkage program significantly enhanced blood glucose management and reduced hypoglycemic events in elderly T2DM patients. These results indicate the significant benefits of informatization in blood glucose management and highlight its potential for broader implementation in clinical practice to enhance patient outcomes.

## 1 Introduction

The prevalence of type 2 diabetes mellitus (T2DM) has become a significant global health concern, particularly affecting the elderly population who were often more vulnerable to its complications (1). According to data from the International Diabetes Federation (IDF), approximately 589 million adults worldwide had diabetes, with China being one of the countries with the highest number of diabetes patients (2). Data from 2023 indicated that the age-standardized overall prevalence of diabetes in China reached 13.7%, with a total of 233 million patients, accounting for one-quarter of all diabetes patients globally (3). This means that one in every six Chinese individuals had diabetes. Compared to 2005, the number of diabetes patients in China increased by 163%, highlighting the severe nature of the domestic diabetes epidemic. Without effective intervention measures, the prevalence of diabetes in China could rise to 29.1% by 2050, posing a significant challenge to the public health system. As life expectancy increases, the number of elderly individuals living with diabetes continues to rise, presenting substantial challenges in disease management (4, 5).

Elderly patients with diabetes were prone to hypoglycemia due to a combination of physiological, pharmacological, behavioral, and cognitive factors. As patients aged, their liver and kidney functions declined, which slowed the clearance of insulin and sulfonylureas and led to drug accumulation (6). Simultaneously, these patients had insufficient secretion of counter-regulatory hormones and reduced ability to perceive and respond to hypoglycemia, making them more susceptible to asymptomatic hypoglycemia. The use of drug treatment regimens, particularly insulin and insulin secretagogues, without individualized adjustments significantly increased the risk (7). Additionally, patients might experience medication errors or inadequate self-monitoring due to cognitive decline or vision problems. Irregular eating habits and exercise further exacerbated blood glucose fluctuations (8). A lack of awareness about hypoglycemia often resulted in delayed intervention, allowing events to progress to severe hypoglycemia.

Hypoglycemia, a common and potentially dangerous complication of diabetes treatment, particularly impacts this demographic, exacerbating comorbid conditions and adversely affecting quality of life (9, 10). In elderly patients with diabetes in China, strict control of hyperglycemia is often accompanied by a higher risk of hypoglycemia. Studies have shown that among elderly patients in China using insulin or sulfonylureas, approximately 40% experience at least one mild hypoglycemic event annually, while 5%–10% encounter severe hypoglycemia (11). Hypoglycemia can lead to symptoms such as palpitations and confusion and is also associated with an increased incidence of cardiovascular events and fall-related injuries. It may even contribute to cognitive dysfunction and an elevated risk of death (12). Therefore, adopting comprehensive management strategies, including individualized medication adjustments, continuous glucose monitoring, patient education, and collaboration among hospitals, communities, and families, was essential for significantly reducing the risk of hypoglycemia, enhancing treatment safety, and improving health outcomes and quality of life in elderly patients with diabetes.

Traditional diabetic management models predominantly focus on pharmacologic interventions and patient education without fully integrating the psychosocial and community dimensions of health care that were essential for effective chronic disease management. This fragmented approach often results in suboptimal diabetes control and increased episodes of hypoglycemia, thereby highlighting the need for a more comprehensive strategy (13–15). In recent years, although both domestic and international studies have increasingly focused on the application of multidisciplinary collaboration in diabetes management, most interventions were still limited to single healthcare institutions or community levels, lacking a systematic hospital-community-family tripartite collaboration mechanism (16). Previous studies did not fully integrate information technology for continuous dynamic management. Comprehensive research targeting hypoglycemia prevention in elderly populations was also limited and often lacked outcome evaluations oriented toward value-based healthcare.

Recent advances in healthcare emphasize value-based medicine, which prioritizes patient outcomes over volume of services provided. This paradigm shift from fee-for-service to value-based medicine mandates the development of models that not only manage the physical symptoms of diabetes but also incorporate elements of community support, family involvement, and holistic patient education to achieve superior clinical outcomes. The inherent complexity of diabetes management in elderly patients necessitates a multifaceted approach that addresses not only the medical but also the behavioral and social determinants of health (17–19).

Recognizing the complexities and limitations of current management approaches, this study aimed to develop an integrated hospital-community-family framework based on value-based healthcare principles. The goal was to address fragmented healthcare resources, lack of continuous patient support, and insufficient self-management capabilities. By using technologies such as electronic health records, remote monitoring, and mobile apps, the study enabled real-time data sharing and multi-party collaboration, providing continuous and personalized patient support. The study evaluated the model’s effectiveness in reducing hypoglycemic events, improving blood glucose control, and enhancing the quality of life for elderly patients with type 2 diabetes, providing practical evidence for value-based healthcare focused on health outcomes.

## 2 Materials and methods

### 2.1 Research design and theoretical basis

This study was a randomized controlled trial. We used a computer-generated random number table to allocate eligible participants in a 1:1 ratio to either the integrated management group or the usual management group. The random allocation sequence was generated and sealed by an independent researcher not involved in patient recruitment or data collection to ensure allocation concealment.

The design of this intervention program was based on an integrated framework of social cognitive theory and the chronic management model (20). Social cognitive theory emphasizes the dynamic interaction between individuals, behaviors, and environments. In this study, “individual” factors focused on enhancing patients’ self-efficacy through interventions, including their knowledge, skills, and confidence in managing diabetes; “behavior” encompassed daily self-management activities such as medication adherence, blood glucose monitoring, diet, and exercise; and “environment” referred specifically to the supportive network built by hospitals, communities, and families. This theory suggested that systematic optimization of environmental support (i.e., tertiary integration) and enhancement of individual capacity (i.e., increasing self-efficacy) could effectively promote patients’ adoption and maintenance of healthy behaviors, leading to better clinical outcomes (21). Additionally, the chronic management model provided structural guidance from the healthcare system perspective, advocating for a community-based, patient-centered management system emphasizing planned follow-ups and multi-role collaboration (22). The “hospital-community-family” integration implemented in this study was a specific application of this model in the context of local primary care.

Based on these theories, we constructed the core conceptual framework of our study. This framework explained how intervention measures influenced final outcomes through key mediating mechanisms. The integrated management program functioned through its core components, such as personalized health records, community education, regular follow-ups, and home visits. These components empowered patients, enhancing their self-management efficacy (patient-level mediating mechanism), while also improving the continuity and coordination of medical services through information platforms and standardized processes (system-level mediating mechanism). Enhanced self-efficacy and improved system coordination together promoted better self-management behaviors (behavioral mechanism), leading to more stable blood glucose control and allowing clinicians to optimize or even reduce antidiabetic medication dosages more precisely (biomedical mechanism). This series of chain reactions ultimately resulted in comprehensive benefits, including improved blood glucose control, reduced hypoglycemic events, and decreased anxiety levels. This conceptual framework provided a rigorous logical basis for selecting intervention measures and determining outcome indicators, ensuring the scientific rigor of the study design.

### 2.2 Participants

From September 2023 to September 2024, we consecutively recruited eligible elderly patients with type 2 diabetes from the Zhangjiagang Sixth People’s Hospital. Inclusion criteria were as follows: participants were eligible if they were over 60 years old, diagnosed with type 2 diabetes according to the guidelines of the Chinese Society for Metabolic and Bariatric Surgery (CSMBS) on “Diagnosis and Treatment Guidelines for Obesity and Type 2 Diabetes” (23), were currently receiving treatment, had complete medical records, were mentally clear, and had normal cognitive function. Exclusion criteria included individuals with respiratory diseases or other infectious diseases, malignancies, severe organ dysfunction, or severe diabetic complications. These were defined as severe renal insufficiency with an estimated glomerular filtration rate (eGFR) < 30 mL/min/1.73 m^2^; unstable angina, myocardial infarction, revascularization, or stroke within the past 6 months; proliferative diabetic retinopathy or macular edema requiring urgent surgery or laser treatment; and severe diabetic peripheral neuropathy or diabetic foot presenting with rest pain, ulcers, or gangrene. Additionally, patients undergoing treatment for severe conditions that could affect glucose management outcomes were also excluded. To control for the influence of confounding factors, patients who were participating in other clinical trials or receiving other intervention measures were also excluded.

**FIGURE 1:**
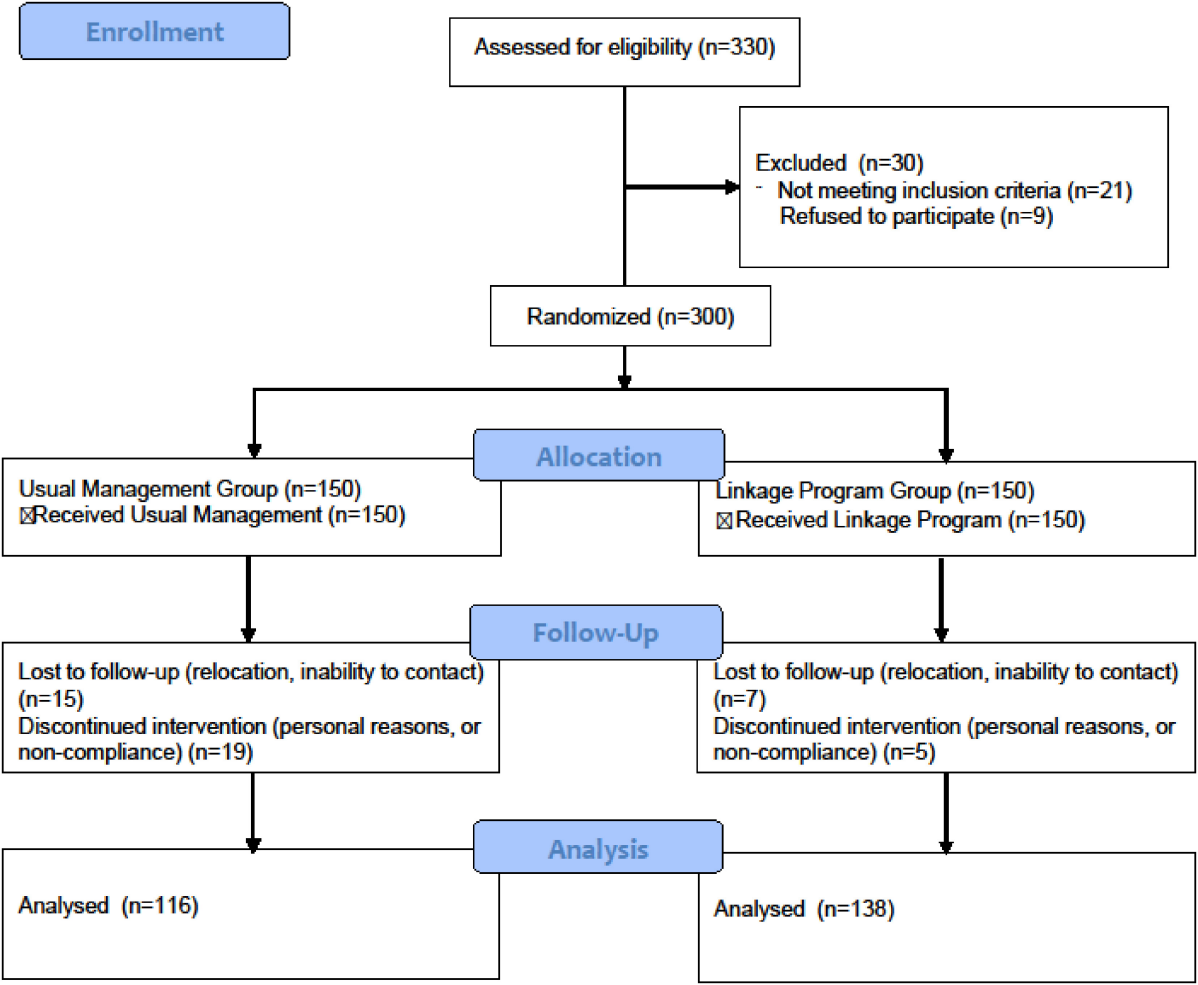
Patient flow diagram.

This study strictly adhered to the standard procedures of a randomized controlled trial (Figure 1). Between September 2023 and September 2024, a total of 330 patients were assessed for eligibility. Among them, 30 patients were excluded, primarily due to not meeting the inclusion criteria (*n* = 21) and refusal to participate (*n* = 9). Ultimately, 300 patients completed randomization and were assigned to either the conventional management group (*n* = 150) or the linkage program group (*n* = 150). During the follow-up period, 15 patients in the conventional management group were lost to follow-up (due to relocation, inability to contact), and 19 patients discontinued the intervention (due to personal reasons, or non-compliance with the intervention protocol). In the linkage program group, 7 patients were lost to follow-up and 5 patients discontinued the intervention. Consequently, data from 116 patients in the conventional management group and 138 patients in the linkage program group were included in the final analysis.

The sample size for this study was estimated using G*Power 3.1.9.7 software. Based on the study design, we selected an *a priori* analysis type of *t*-test (independent groups, two-tailed). The parameters were set as follows: a medium effect size d of 0.5, an α error probability of 0.05, and a statistical power (1−β) of 0.95. The calculation indicated that the minimum required sample size per group was 105 participants, resulting in a total of at least 210 participants for both groups combined. To account for an anticipated dropout or data loss rate of approximately 15% during the study, we increased the total sample size to 300 participants. Ultimately, this study included and completed randomization and data analysis for 254 patients, with 138 in the integrated management group and 116 in the usual management group. The actual sample size slightly exceeded the estimated value, ensuring sufficient statistical power for the study.

### 2.3 Ethical considerations

The study adhered to the guidelines outlined in the Declaration of Helsinki and received approval from the Institutional Review Board of the Zhangjiagang Sixth People’s Hospital. The study was approved by the Medical Ethics Committee of Zhangjiagang Sixth People’s Hospital (Ethics Approval Number: ZJGLY-LW-20230601). Written informed consent was provided by all participants.

### 2.4 Management plan

#### 2.4.1 Conventional management

The conventional management plan involved systematic health education on diabetes, dietary guidance, medication instruction, and self-monitoring techniques. Upon discharge, patients were expected to understand the relevant educational points and maintain a focus on their diet. In everyday life, patients were encouraged to ensure adequate sleep, wear loose and comfortable clothing, keep warm, and engage in a certain amount of aerobic exercise.

#### 2.4.2 Value-based “hospital-community-family” integrated management

The value-based “hospital-community-family” integrated management was a 6-months structured intervention designed and implemented according to the guidance of social cognitive theory and the chronic management model. This program aimed to create a continuous and supportive management environment for patients through systematic collaboration at the hospital, community, and family levels. The entire intervention process consisted of six core components.

The intervention began with the hospital initiation phase. After patient admission, endocrinologists and diabetes specialist nurses completed personalized assessments within 24 h using an electronic health record system to collect comprehensive data on the patient’s condition, complications, self-management abilities, and family support status. Based on this assessment, doctors and patients jointly developed an “Individualized Diabetes Health Management File” before discharge, which outlined individualized blood glucose targets, dietary and exercise plans, and medication strategies. During their hospital stay, patients received three structured education sessions, each lasting 45–60 min, led by diabetes specialist nurses in group settings. These sessions used standardized health manuals and video materials to systematically cover diabetes basics, hypoglycemia recognition and prevention, correct medication use, proper glucose meter operation, foot care, and emergency procedures.

After discharge, the intervention transitioned to the community linkage and family support phase, which continued until the study ended. At the community level, trained general practitioners or nurses conducted biweekly phone follow-ups, each lasting 15–24 min. Follow-ups followed a standardized checklist to track patients’ blood glucose monitoring data, medication adherence, diet and exercise compliance, and hypoglycemic events. They also provided professional answers to patients’ questions and immediate encouragement for good adherence. All follow-up records were entered into the regional health information platform in real time. Additionally, communities organized monthly “Diabetes Patient Support Group” meetings, each lasting 60 min, supervised by hospital specialists and coordinated by community healthcare workers. These meetings included expert lectures and peer experience sharing, along with demonstrations of suitable exercises such as brisk walking and tai chi.

At the family level, standardized home visits were a crucial component of the intervention. Specialist nurses conducted these visits 1 month and 3 months after discharge. The visits assessed patients’ physical health and blood glucose levels, evaluated home safety and medication storage conditions, and verified proper glucose monitoring techniques. Each visit included focused training for a primary family caregiver on recognizing hypoglycemia symptoms, assisting with medication supervision, and supporting healthy meal preparation to ensure effective family support. Data collected during home visits were synchronized to the central electronic health record system via mobile terminal apps.

Information technology support served as a core measure throughout the intervention, linking all components. Patients received standardized smart glucometers that automatically uploaded measurement data to the information platform. The platform featured an automatic alert system that sent notifications to responsible specialist doctors and specialist nurses upon detecting sustained hyperglycemia or suspected hypoglycemia, triggering timely proactive interventions. This interconnected intervention process ensured continuous management from hospital to community to home throughout the patient’s course of illness.

### 2.5 Outcome measures

Data were extracted from electronic medical records and encompassed demographic characteristics, baseline disease-related features, outcomes and blood glucose level. Specific parameters recorded included age, Body Mass Index (BMI), smoking and drinking status, duration of diabetes, and medication regimens. Throughout the study period, blood glucose levels, hypoglycemic events, and adverse events were carefully monitored. The normal range for adult FPG was 3.9–6.1 mmol/L, the standard range for 2 h PG was 4.4–7.8 mmol/L.

#### 2.5.1 Primary outcome measures

The incidence of hypoglycemic events is the primary safety and efficacy measure of this study. We recorded the total number of hypoglycemic events per month for each participant (defined as blood glucose < 3.9 mmol/L) and further categorized them into nocturnal hypoglycemia, severe hypoglycemia (requiring assistance from others), and mild hypoglycemia.

Blood glucose control was assessed through fasting blood glucose. This indicator is a core biomedical metric in diabetes management, and its improvement reflects the overall effectiveness of the intervention in stabilizing blood glucose levels. It serves as a critical intermediate link between self-management behaviors and ultimate health outcomes.

#### 2.5.2 Secondary outcome measures

The Diabetes Management Self-Efficacy Scale (DMSES) was employed to assess the confidence levels of patients with T2DM in managing their condition. This study utilized the Chinese version of the DMSES, which has been translated and validated for reliability and validity (24). The scale originated from the Dutch version and was developed based on Bandura’s social cognitive theory. It consists of 20 items. This scale evaluates self-efficacy across various aspects of diabetes management, including dietary control, regular physical activity, medication adherence, and blood glucose monitoring. The total score of the scale was 200, with higher scores indicating greater self-efficacy. The DMSES demonstrates good reliability, with a Cronbach’s α coefficient of 0.93 (25).

We recorded the daily doses of various antidiabetic medications used by patients in each group, including metformin, sulfonylureas, DPP-4 inhibitors, SGLT-2 inhibitors, and insulin. This indicator reflects the treatment intensity required to achieve good blood glucose control after improvements in behavior and system support.

Patients’ anxiety levels were assessed using the State-Trait Anxiety Inventory - State Anxiety Scale (STAI-SA) (26). This scale has a Cronbach’s α coefficient of 0.838 (27), indicating good reliability. The total score ranges from 20 to 80, with scores of 20–39 indicating low anxiety, 40–59 indicating moderate anxiety, and 60–80 indicating high anxiety.

All data related to adverse events were systematically collected and recorded to comprehensively assess the safety of the integrated program.

### 2.6 Statistical methods

Data analysis was conducted using SPSS statistical software version 29.0 (SPSS Inc., Chicago, IL, USA). Continuous variables that followed a normal distribution were presented as mean ± standard deviation (X ± s). Group comparisons for these variables were conducted using independent samples *t*-tests. Categorical variables were described using frequencies and percentages (*n*, %), and group comparisons for these variables were performed using chi-square (χ^2^) tests. A *p*-value of less than 0.05 was considered statistically significant. To further understand the magnitude of differences between groups for various parameters, Cohen’s d effect sizes were calculated.

## 3 Results

### 3.1 Demographic characteristics

The age of participants in the usual management group was 68.78 ± 7.85 years compared to 68.19 ± 8.19 years in the linkage program group (*P* = 0.561) (Table 1). The BMI was similar between the two groups, with the usual management group at 23.62 ± 4.26 kg/m^2^ and the linkage program group at 23.4 ± 4.18 kg/m^2^ (*P* = 0.678). Gender distribution showed 28.45% females in the usual management group and 25.36% in the linkage program group (*P* = 0.681). No significant differences were observed in smoking status, drinking status, physical activity, education level, occupation status, difficulty paying for basics, marital status, monthly average income, or hypertension (*P* > 0.05). These findings indicate comparable baseline characteristics between the groups, allowing for a fair assessment of the program’s impact on hypoglycemia outcomes.

**TABLE 1:** Comparison of demographic characteristics between two groups.

Index	Usual management group (*n* = 116)	Linkage program group (*n* = 138)	t/x^2^	*P*
Age (years)	68.78 ± 7.85	68.19 ± 8.19	0.582	0.561
BMI (kg/m^2^)	23.62 ± 4.26	23.40 ± 4.18	0.416	0.678
Gender (female/male)	33 (28.45%)/83 (71.55%)	35 (25.36%)/103 (74.64%)	0.169	0.681
Smoking status (yes/no)	23 (19.83%)/93 (80.17%)	26 (18.84%)/112 (81.16%)	0.002	0.969
Drinking status (yes/no)	11 (9.48%)/105 (90.52%)	14 (10.14%)/124 (89.86%)	0.031	0.860
Physical activity (hours/week)	4.59 ± 2.14	4.23 ± 1.87	1.417	0.158
Education level (high school and below/bachelor degree and above)	93 (80.17%)/23 (19.83%)	101 (73.19%)/37 (26.81%)	1.339	0.247
Occupation status (working/not working)	49 (42.24%)/67 (57.76%)	52 (37.68%)/86 (62.32%)	0.373	0.541
Difficulty paying for basics (hard/not hard)	71 (61.21%)/45 (38.79%)	78 (56.52%)/60 (43.48%)	0.394	0.530
Marital status (single/married/divorced)	31 (26.72%)/62 (53.45%)/23 (19.83%)	38 (27.54%)/78 (56.52%)/22 (15.94%)	0.660	0.719
Monthly average income (<3000/3000∼6000/>6000)	24 (20.69%)/59 (50.86%)/33 (28.45%)	23 (16.67%)/59 (42.75%)/56 (40.58%)	4.090	0.129
Hypertension (yes/no)	29 (25.00%)/87 (75.00%)	31 (22.46%)/107 (77.54%)	0.106	0.745

BMI, body mass index.

### 3.2 Comparison of primary and secondary outcomes between groups before and after the intervention

The duration of diabetes was similar between the groups, with the usual management group at 9.03 ± 2.28 years and the linkage program group at 8.84 ± 2.59 years (*P* = 0.524) (Table 2). Family history of diabetes was reported in 24.14% of the usual management group and 23.91% of the linkage program group (*P* = 1.000). The use of self-blood glucose monitoring was comparable, with 50.86% in the usual management group and 48.55% in the linkage program group (*P* = 0.810). Although a higher proportion of patients in the linkage program group used only insulin injections (76.09% vs. 64.66%), this difference approached but did not reach statistical significance (*P* = 0.063). Similarly, the combination of oral medicine and insulin injection showed no significant difference (*P* = 0.227). The average daily dose of insulin was also comparable between the groups (42.53 ± 13.89 units vs. 41.46 ± 12.86 units; *P* = 0.528). Staple food control was similarly distributed between groups (*P* = 0.246). These findings suggest that both groups were well matched in terms of baseline disease characteristics.

**TABLE 2:** Comparison of baseline disease-related features between two groups.

Index	Usual management group (*n* = 116)	Linkage program group (*n* = 138)	t/x^2^	*P*
Duration of diabetes (years)	9.03 ± 2.28	8.84 ± 2.59	0.637	0.524
Family history of diabetes (yes/no)	28 (24.14%)/88 (75.86%)	33 (23.91%)/105 (76.09%)	0.002	0.967
Use of SBGM (yes/no)	59 (50.86%)/57 (49.14%)	67 (48.55%)/71 (51.45%)	0.058	0.810
Only insulin injection (yes/no)	75 (64.66%)/41 (35.34%)	105 (76.09%)/33 (23.91%)	3.455	0.063
Oral medicine plus insulin injection (yes/no)	25 (21.55%)/91 (78.45%)	40 (28.99%)/98 (71.01%)	1.459	0.227
The average daily dose of insulin injection (units)	42.53 ± 13.89	41.46 ± 12.86	0.632	0.528
Staple food control (≤400 g/d/>400 g/d)	89 (76.72%)/27 (23.28%)	115 (83.33%)/23 (16.67%)	1.348	0.246

SBMG, self-blood glucose monitoring.

While initial FPG levels were similar between the usual management group and the linkage program group (10.14 ± 1.36 mmol/L vs. 10.12 ± 1.47 mmol/L, *P* = 0.914), post-management FPG was significantly reduced in the linkage program group (6.93 ± 1.3 mmol/L vs. 7.46 ± 1.94 mmol/L, *P* = 0.013) (Table 3). Although the 2-hour postprandial glucose levels decreased more in the linkage program group after management (8.79 ± 2.57 mmol/L vs. 9.44 ± 2.96 mmol/L), this did not reach statistical significance (*P* = 0.064). These results suggest that the linkage program yielded substantial improvements in blood glucose control among participants compared to the usual management approach.

**TABLE 3:** Comparison of blood glucose between two groups of patients.

Index	Usual management group (*n* = 116)	Linkage program group (*n* = 138)	t	*P*
FPG before management (mmol⋅L)	10.14 ± 1.36	10.12 ± 1.47	0.108	0.914
FPG after management (mmol⋅L)	7.46 ± 1.94	6.93 ± 1.30	2.512	0.013
2-h PG before management (mmol⋅L)	12.76 ± 1.49	12.68 ± 1.59	0.405	0.686
2-h PG after management (mmol⋅L)	9.44 ± 2.96	8.79 ± 2.57	1.861	0.064

FPG, fasting plasma glucose; PG, postprandial blood glucose.

**FIGURE 2:**
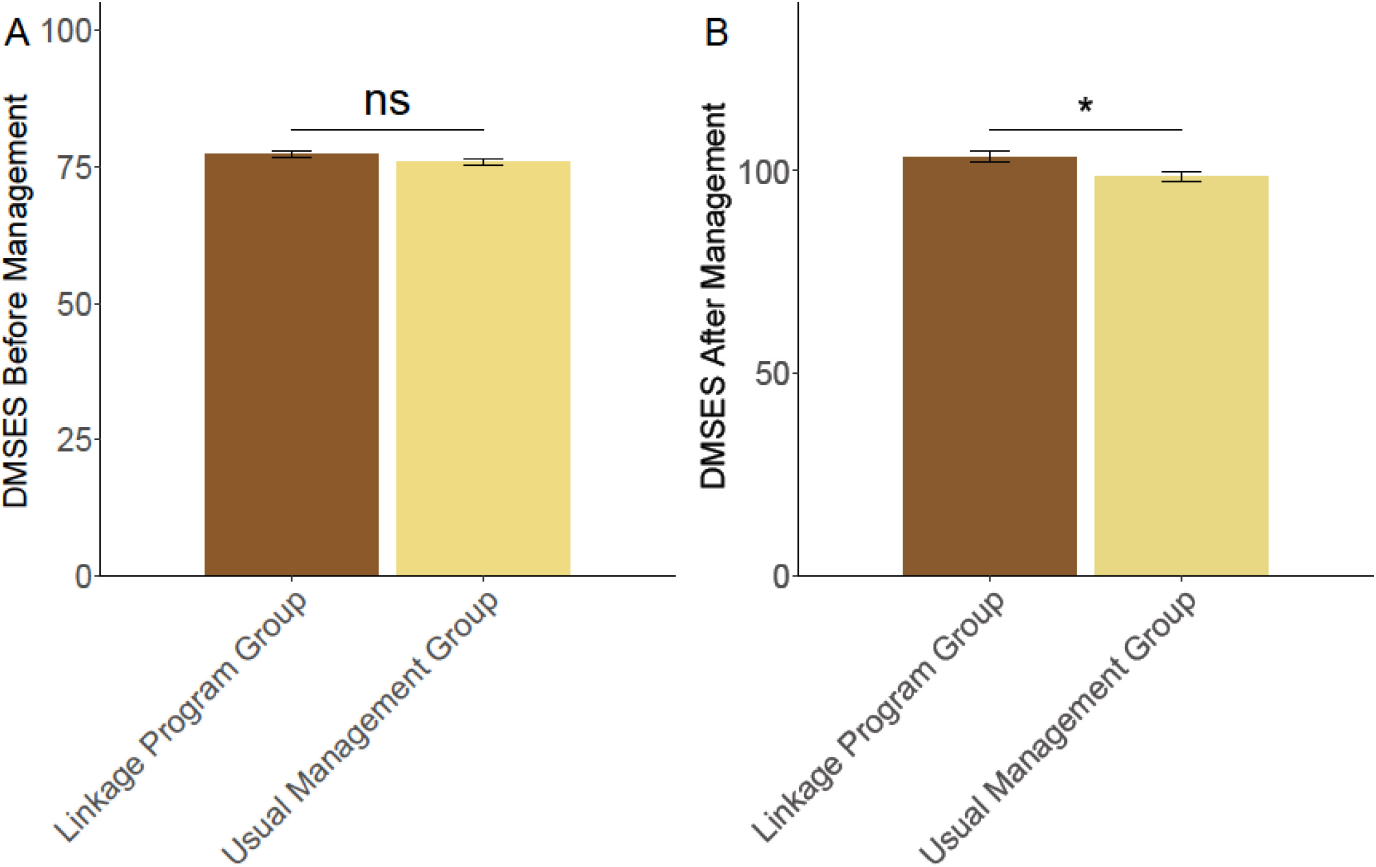
Comparison of DMSES between two groups of patients. **(A)** DMSES before management. **(B)** DMSES after management. DMSES, diabetes management self-efficacy score. Ns, no significant difference; *P < 0.05.

Prior to the management intervention, there was no significant difference in self-efficacy scores between the usual management group and the linkage program group (75.86 ± 5.73 vs. 77.29 ± 5.81, *P* = 0.051) (Figure 2). However, post-management, the linkage program group demonstrated a significantly higher self-efficacy score compared to the usual management group (103.47 ± 15.53 vs. 98.59 ± 14.16, *P* = 0.009). This suggests that participation in the linkage program was associated with a significant enhancement in patients’ confidence in managing their diabetes.

Dizziness occurred in 9.48% of the usual management group compared to 6.52% in the linkage program group (*P* = 0.523) (Table 4). Gastrointestinal issues were reported by 11.21% of participants in the usual management group and 7.25% in the linkage program group (*P* = 0.381). The incidence of skin reactions was 4.31% in the usual management group and 2.90% in the linkage group (*P* = 0.791). Fatigue was more frequent in the usual management group (13.79%) than in the linkage program group (5.80%), but this difference was not statistically significant (*P* = 0.051). Cases of allergic reactions, edema, cardiovascular events, and neurological symptoms were low and similar between the two groups, with no significant differences (*P* > 0.05). These findings indicate that participation in the linkage program did not lead to an increased risk of adverse events compared to usual management.

**TABLE 4:** Adverse events among participants.

Parameters	Usual management group (*n* = 116)	Linkage program group (*n* = 138)	χ ^2^	*P*
Dizziness (%)	11 (9.48%)	9 (6.52%)	0.408	0.523
Gastrointestinal issues (%)	13 (11.21%)	10 (7.25%)	0.768	0.381
Skin reactions (%)	5 (4.31%)	4 (2.90%)	0.071	0.791
Fatigue (%)	16 (13.79%)	8 (5.80%)	3.821	0.051
Allergic reactions (%)	4 (3.45%)	3 (2.17%)	0.054	0.816
Edema (%)	5 (4.31%)	4 (2.90%)	0.071	0.791
Cardiovascular events (%)	7 (6.03%)	8 (5.80%)	0.000	1.000
Neurological symptoms (%)	6 (5.17%)	4 (2.90%)	0.365	0.546

**FIGURE 3:**
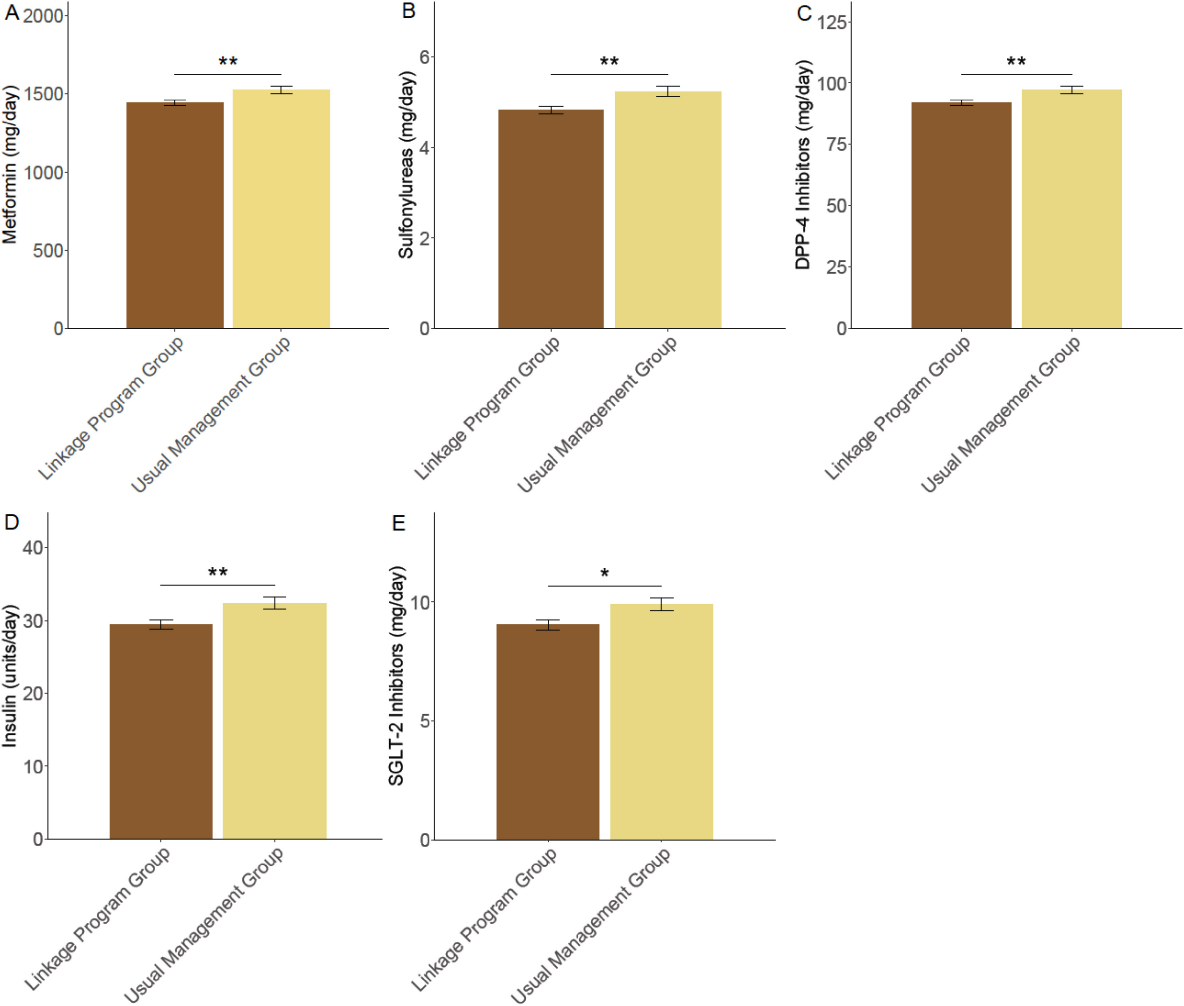
Therapeutic drug doses. **(A)** Metformin (mg/day); **(B)** Sulfonylurea (mg/day); **(C)** DPP-4 inhibitors (mg/day); **(D)** Insulin (units/day); **(E)** SGLT-2 inhibitors (mg/day). *P < 0.05; **P < 0.01.

The average daily dosage of metformin was significantly lower in the linkage program group (1441.66 ± 221.11 mg) compared to the usual management group (1523.47 ± 251.02 mg, *P* = 0.007) (Figure 3). Similarly, participants in the linkage program required a lower dose of sulfonylureas (4.83 ± 1.01 mg) than those in the usual management group (5.24 ± 1.21 mg, *P* = 0.004). The dosage of DPP-4 inhibitors was reduced in the linkage program group (91.87 ± 12.42 mg) compared to the usual management group (97.11 ± 15.17 mg, *P* = 0.003). Insulin requirements were also less in the linkage program group (29.44 ± 7.03 units/day) versus the usual management group (32.37 ± 8.54 units/day, *P* = 0.004). Additionally, a reduction in the dose of SGLT-2 inhibitors was observed in the linkage program group (9.05 ± 2.48 mg) compared to the usual management group (9.91 ± 2.84 mg, *P* = 0.011). These findings indicate that the linkage program effectively reduced the need for higher drug doses, suggesting improved glycemic control and therapeutic efficiency.

A significantly higher proportion of patients in the linkage program group reported low anxiety levels (20–39 points) at 88.41%, compared to 73.28% in the usual management group (Table 5). On the other hand, the incidence of moderate anxiety (40–59 points) was lower in the linkage program group at 5.07% compared to 16.38% in the usual management group. High anxiety levels (60–80 points) were observed in 6.52% of the linkage program group, slightly less than the 10.34% in the usual management group. The overall difference in anxiety levels between the two groups was statistically significant (χ^2^ = 10.756, *P* = 0.005), indicating that participation in the linkage program was associated with significantly lower anxiety among elderly diabetic patients.

**TABLE 5:** Comparison of STAI-SA score between the two groups.

Parameters	Usual management group (*n* = 116)	Linkage program group (*n* = 138)	t/χ ^2^	*P*
Low anxiety (20–39 points)	85 (73.28%)	122 (88.41%)	10.756	0.005
Moderate anxiety (40–59 points)	19 (16.38%)	7 (5.07%)		
High anxiety (60–80 points)	12 (10.34%)	9 (6.52%)		

**FIGURE 4:**
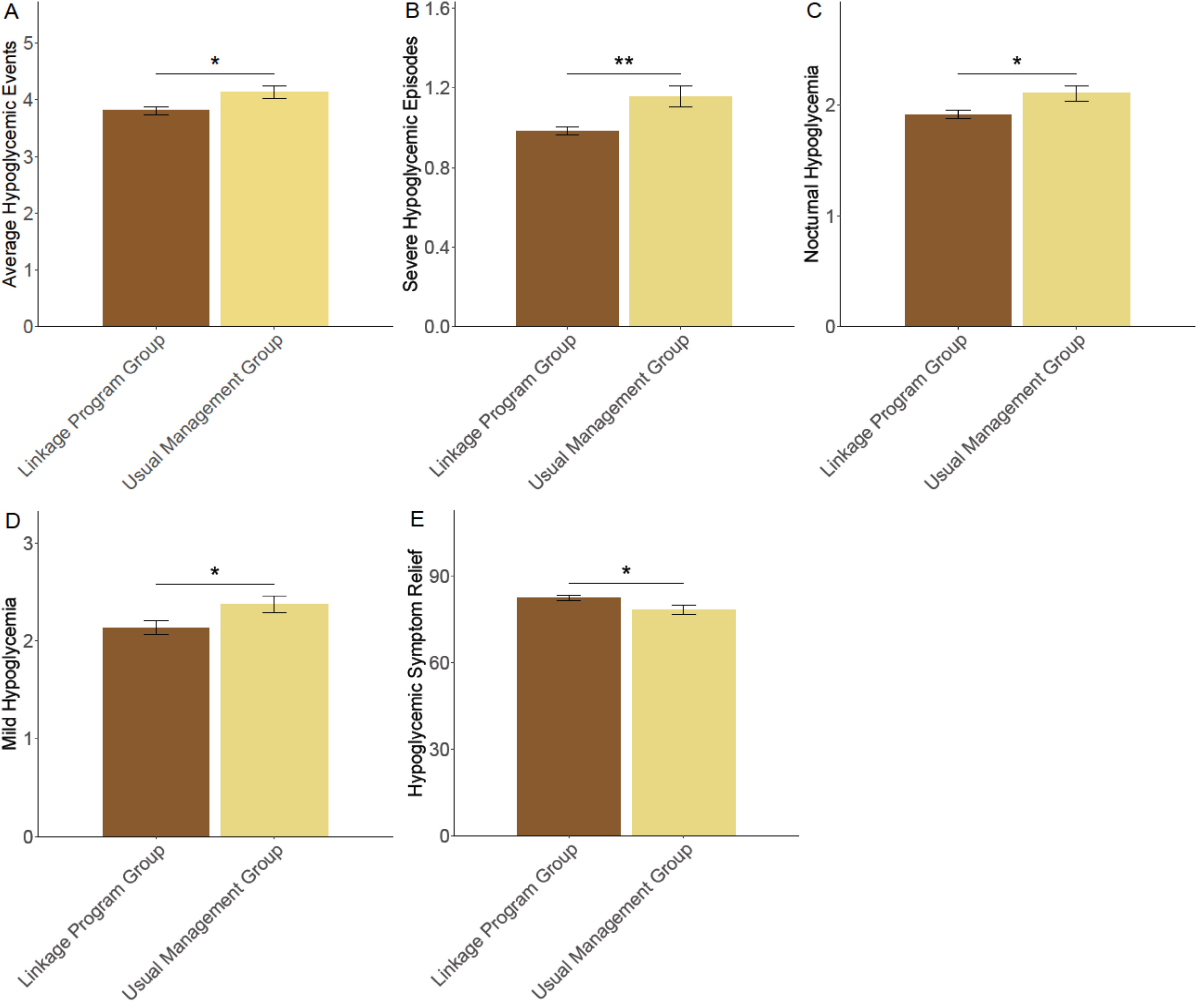
Incidence of hypoglycemic events (events/month). **(A)** Average hypoglycemic events; **(B)** severe hypoglycemic episodes; **(C)** nocturnal hypoglycemia; **(D)** mild hypoglycemia; **(E)** hypoglycemic symptom relief. *P < 0.05; **P < 0.01.

The average number of hypoglycemic events per month was significantly lower in the linkage program group (3.81 ± 0.89) than in the usual management group (4.13 ± 1.17, *P* = 0.015) (Figure 4). Participants in the linkage program also experienced fewer severe hypoglycemic episodes (0.98 ± 0.25 vs. 1.16 ± 0.56, *P* = 0.002). Nocturnal hypoglycemia was reduced in the linkage program group (1.91 ± 0.44) compared to the usual management group (2.11 ± 0.72, *P* = 0.013). Additionally, there was a decrease in mild hypoglycemic episodes in the linkage program group (2.13 ± 0.84) compared to the usual management group (2.37 ± 0.91, *P* = 0.032). Furthermore, participants in the linkage program reported improved relief from hypoglycemic symptoms (82.41 ± 10.02) compared to those in the usual management group (78.23 ± 18.18, *P* = 0.028). These findings suggest that the linkage program was effective in reducing the incidence and severity of hypoglycemic events in elderly diabetic patients.

### 3.3 Effect size analysis

The Cohen’s d effect sizes indicate the magnitude of differences between groups for various parameters (Table 6). Most demographic and baseline characteristics such as age, BMI, duration of diabetes, and average daily dose of insulin injection showed very small effect sizes, suggesting minimal differences between groups. Similarly, pre-management factors like FPG before management, 2-h PG before management, and HbA1c before management also had negligible effect sizes. Post-management, however, several parameters demonstrated moderate to large effect sizes, indicating more substantial differences between groups. Notably, FPG after management (0.327), 2-h PG after management (0.237), HbA1c after management (0.375), severe hypoglycemic episodes (0.415), and medication dosages including Metformin (0.348), Sulfonylureas (0.370), DPP-4 inhibitors (0.382), Insulin (0.378), and SGLT-2 inhibitors (0.326) all showed moderate to large effect sizes. Additionally, self-efficacy scores, specifically DMSES before management (−0.247) and after management (−0.327), along with hypoglycemic symptom relief (−0.292), exhibited negative effect sizes, indicating a reduction in these measures in one group compared to the other.

**TABLE 6:** Cohen’s d effect sizes.

Parameters	Cohen’s d
Age (years)	0.073
BMI (kg/m^2^)	0.052
Physical activity (hours/week)	0.181
Duration of diabetes (years)	0.079
The average daily dose of insulin injection (units)	0.080
FPG before management (mmol⋅L)	0.014
FPG after management (mmol⋅L)	0.327
2-h PG before management (mmol⋅L)	0.051
2-h PG after management (mmol⋅L)	0.237
HbA1c before management (%)	0.138
HbA1c after management (%)	0.375
Diabetes management self-efficacy score (DMSES) before management	−0.247
Diabetes management self-efficacy score (DMSES) after management	−0.327
Average hypoglycemic events	0.316
Severe hypoglycemic episodes	0.415
Nocturnal hypoglycemia	0.330
Mild hypoglycemia	0.274
Hypoglycemic symptom relief	−0.292
Metformin (mg/day)	0.348
Sulfonylureas (mg/day)	0.370
DPP-4 inhibitors (mg/day)	0.382
Insulin (units/day)	0.378
SGLT-2 inhibitors (mg/day)	0.326

## 4 Discussion

The study aimed to evaluate the impact of a hospital-community-family linkage blood glucose management program based on value-based medicine on hypoglycemia and associated outcomes in elderly patients with type 2 diabetes. One of the main findings of this study was the significant reduction in hypoglycemic events among patients participating in the linkage program. This outcome can be attributed to several interrelated factors within the integrated management approach and informatization. On using information technology solutions such as electronic health records, remote monitoring devices, and mobile applications, hospitals, communities, and families were able to collaborate more effectively, ensuring regular monitoring and timely interventions, thereby preventing glucose fluctuations that lead to hypoglycemia (28, 29). Informatization enabled real-time data sharing, enhancing communication efficiency among healthcare providers at various levels, ensure that patients’ conditions were continuously monitored and that any deviations from the norm were quickly addressed (30–32). This vigilance was essential in preventing both mild and severe hypoglycemic episodes, which were particularly concerning in the elderly due to their potential to cause acute health crises.

Another contributing factor to the observed reduction in hypoglycemic events could be the improvement in patients’ self-management abilities. As evidenced by the study, participants in the integrated program exhibited higher diabetes management self-efficacy scores post-intervention. The application of informatization not only broadened the scope of educational efforts but also enhanced patients’ self-management confidence, which may have resulted from the comprehensive educational initiatives implemented, including community-wide education activities and the dissemination of materials focused on diabetes management. This education enabled patients to make informed decisions regarding their medication, diet, and lifestyle, thereby reducing the likelihood of hypoglycemia events (33–35). Additionally, the program’s emphasis on teaching the proper use of glucometers and medication compliance ensures that patients can effectively monitor and adjust their blood glucose levels independently, providing another layer of prevention against hypoglycemia.

The linkage program’s impact on medication dosage was also a major consideration. Participants in the linkage program required significantly lower doses of hypoglycemic agents, including metformin, sulfonylureas, DPP-4 inhibitors, insulin, and SGLT-2 inhibitors. This indicates that, with the support of informatization, the integrated management approach facilitated better blood glucose control and achieved optimal treatment outcomes with less reliance on pharmacological interventions. Lower medication doses were advantageous as they reduce the risk of drug-related adverse events, including hypoglycemia, which was a known side effect of various antidiabetic medications (36–38). The ability to achieve glycemic targets with reduced drug dependency highlights the effectiveness of a holistic management strategy that considers the interaction between medications, lifestyle modifications, and continuous blood glucose management.

Furthermore, the study demonstrated significant improvements in FPG and glycated hemoglobin levels in the linkage program group compared to the usual management group. These improvements reflect superior glycemic control achieved through the comprehensive and continuous management model. Informatization not only facilitates the automatic collection and analysis of data but also enhances the accuracy and timeliness of data through automated reporting functions. The program likely induces behavioral modifications conducive to long-term glycemic stability through the involvement of multiple management delivery levels and a strong emphasis on consistent patient engagement. In this study, the improvement in 2-hour postprandial blood glucose did not reach statistical significance. This might have been because the intervention focused on preventing hypoglycemia and stabilizing fasting blood glucose, without placing enough emphasis on detailed management of postprandial carbohydrate intake, insulin dosage, and post-meal activities. Additionally, variations in how elderly patients managed their postprandial routines contributed to the lack of observed differences in 2-hour postprandial glucose levels between groups. The role of family members, as part of the linkage program, cannot be overlooked. Their involvement in regularizing medication, guiding diet and rest, and providing emotional support creates a supportive environment that fosters adherence to diabetes management plans and promotes healthier lifestyle choices (39, 40).

An interesting observation in this study was the significant reduction in anxiety levels among participants in the linkage program. Given that psychological stress can adversely affect glycemic control by triggering counter-regulatory hormonal responses, reducing anxiety may directly contribute to better diabetes management outcomes. The comprehensive support system offered by the program, including psychological counseling and active doctor involvement, likely alleviates anxiety by providing reassurance and addressing any concerns that patients might have about their condition and treatment (41). A reduction in anxiety can lead to improved compliance with self-care tasks and medication regimens, indirectly contributing to reductions in hypoglycemic events and better overall metabolic control (42).

The success of this study can be attributed to the successful establishment of a patient-centered, information-driven, and multi-party collaborative continuous management system. The reduction in hypoglycemic events is due to the “full-process warning and proactive intervention” capabilities provided by smart glucose meters and information platforms, enabling preventive rather than reactive management. The precise adjustment of medication doses and concurrent improvement in blood glucose control reflect significant enhancements in patients’ self-management behaviors through structured education in hospitals, community follow-ups, and home visit training. The notable decrease in anxiety levels is attributed to the continuous and reliable support system provided by this model, which enhances patients’ psychological sense of security, alleviating disease uncertainty and treatment-related fears.

Compared to previous studies, this research stands out by integrating value orientation, tertiary-level collaboration, and information technology. Prior studies often lacked data connectivity or were limited to a single level, resulting in poor continuity post-discharge. This study ensures seamless integration of patient data and interventions among hospitals, communities, and families via a unified platform, enhancing management continuity and coordination. This leads to broader coverage and greater precision, significantly improving hypoglycemia management. Additionally, by considering antidiabetic medication doses as a secondary outcome, it evaluates the potential to reduce healthcare costs from a “value-based management” perspective, adding a distinctive and forward-looking dimension.

The intervention plan was designed with consideration for economic feasibility, avoiding the introduction of expensive new equipment or medications. Instead, it focuses on optimizing the allocation and efficiency of existing medical resources. Key measures include the use of mature smart glucose meters and regional health information platforms, keeping incremental costs manageable. This plan aligns with China’s healthcare system reform, which is based on primary care, and emphasizes the central role of families in elderly management. By activating the potential of community health service centers and integrating family members into the management team, this model ensures the sustainability and replicability of the intervention.

However, despite the promising results, the study was not without limitations. The data were extracted from a specific community hospital setting, and the results might not be generalizable to other populations or healthcare systems with different resources and structures. Social desirability bias may exist in this study. Because participants are aware that they are part of a special management program, they might be inclined to report self-management behaviors, hypoglycemic events, or complete anxiety scales in a manner that aligns more closely with socially desirable responses. This tendency could potentially overestimate the intervention’s effectiveness. Additionally, although our study provides preliminary evidence supporting the effectiveness of a value-based integrated management approach, the relatively small sample size limits the robustness of our conclusions. A larger sample size would provide richer data and help more accurately estimate the intervention effects. Future research should aim to replicate these findings in diverse settings, potentially through randomized controlled trials, to validate the efficacy of the integrated value-based management approach.

## 5 Conclusion

In conclusion, the hospital-community-family linkage program represents a significant advancement in diabetes management for the elderly, combining medical science with a value-based approach to achieve superior outcomes in hypoglycemia management and overall patient well-being. The program sets a benchmark for comprehensive chronic disease management by addressing the multidimensional aspects of diabetes management, including medical treatment, psychological well-being, and family involvement. The findings highlight the importance of an integrated management model in achieving better health outcomes and enhancing the quality of life for elderly diabetic patients, making a strong case for broader implementation in clinical practice. In special, integrating informatization into therapy practices has proven essential for optimizing patient management and supporting broader implementation in clinical practice.

## Data Availability

The original contributions presented in this study are included in this article/Supplementary material, further inquiries can be directed to the corresponding author.
